# Abnormal band bowing effects in phase instability crossover region of GaSe_1-*x*_Te_*x*_ nanomaterials

**DOI:** 10.1038/s41467-018-04328-z

**Published:** 2018-05-15

**Authors:** Hui Cai, Bin Chen, Mark Blei, Shery L. Y. Chang, Kedi Wu, Houlong Zhuang, Sefaattin Tongay

**Affiliations:** 1 School for Engineering of Matter, Transport and Energy, Arizona State University, Tempe, AZ 85287 USA; 2 LeRoy Eyring Center for Solid State Science, Arizona State University, Tempe, AZ 85287 USA

## Abstract

Akin to the enormous number of discoveries made through traditional semiconductor alloys, alloying selected 2D semiconductors enables engineering of their electronic structure for a wide range of new applications. 2D alloys have been demonstrated when two components crystallized in the same phase, and their bandgaps displayed predictable monotonic variation. By stabilizing previously unobserved compositions and phases of GaSe_1−*x*_Te_*x*_ at nanoscales on GaAs(111), we demonstrate abnormal band bowing effects and phase instability region when components crystallize in different phases. Advanced microscopy and spectroscopy measurements show as tellurium is alloyed into GaSe, nanostructures undergo hexagonal to monoclinic and isotropic to anisotropic transition. There exists an instability region (0.56 < *x* < 0.67) where both phases compete and coexist, and two different bandgap values can be found at the same composition leading to anomalous band bowing effects. Results highlight unique alloying effects, not existing in single-phase alloys, and phase engineering routes for potential applications in photonic and electronics.

## Abstract (web summary)

Alloys of two-dimensional materials normally occur when two components crystallize in the same phase. Here, the authors observe an anomalous phase instability, accompanied by a band bowing effect, in GaSe_1-*x*_Te_*x*_ alloys on GaAs(111).

## Introduction

Two-dimensional (2D) semiconductors not only share many of the superb properties of graphene, but also offer natively semiconducting functions that graphene lacks^1, 2^. Akin to the enormous number of discoveries made through traditional semiconductor alloys (e.g., Al_*x*_Ga_1−*x*_As, In_*x*_Ga_1−*x*_N, CuIn_*x*_Ga_1−*x*_Se_2_), alloying selected 2D semiconductors enables engineering of their electronic structure for a wide range of new applications. In the light of this, much effort has been given to 2D alloys to achieve continuous bandgap tunability across the composition range. Similar to traditional approaches taken for bulk (3D) Al_*x*_Ga_1−*x*_As^3^ and Zn_*x*_Mg_1−*x*_O^4^ semiconductors, a number of 2D alloys has been synthesized and investigated by alloying different chalcogens (MoS_2*x*_Se_2−2*x*_, WS_2*x*_Se_2−2*x*_)^5–11^ or transition metals (Mo_*x*_W_1−*x*_S_2_, Mo_*x*_W_1−*x*_Se_2_)^12–17^.

These studies, however, were mostly restricted to alloying chalcogen or metal atoms and in the entire composition range alloyed materials remained absolutely in the same phase. Taking Mo_*x*_W_1−*x*_Se_2_ as an example, both WSe_2_ (*x* = 0) and MoSe_2_ (*x* = 1) are crystallized in hexagonal (2H-) phase in their energetically most stable configuration, thus same phase is stabilized and experimentally observed when Mo is alloyed into WSe_2_ across the entire composition range. These fixed phase 2D ternary alloy systems follow the fundamentals of alloying theory wherein the optical and electronic bandgap values continuously span across the composition range with variety of band bowing parameters specific to material system and atom types^18^. The band-gap variation with the composition does not always follow a linear relationship instead shows some deviation which is defined and quantified by the band bowing parameter^3^.

Large band bowing and full spectrum band variation is typically observed in highly mismatched alloys such as GaN_*x*_As_1−*x*_^19, 20^ and ZnO_*x*_Te_1−*x*_^21, 22^ wherein alloyed atoms possess large difference in atomic radius and electronegativity. Large difference in radius and electronegativity, in return, limits our ability to attain alloying across the entire composition range. In contrast, it is easier to synthesize an attain full composition coverage for small mismatch alloys, however this also leads to small band bowing parameter and limited band-gap tunability.

Here we report on unique phase instability cross-over and anomalous band bowing effects in GaSe_1−*x*_Te_*x*_ ternary alloys by changing the composition range across isotropic hexagonal GaSe to anisotropic monoclinic GaTe nanomaterials. Prior studies by our team and others have successfully demonstrated that GaSe (*x* = 0) and GaTe (*x* = 1) crystallize in completely different isotropic 2D hexagonal and anisotropic 2D monoclinic phases, respectively^23–30^. We find that GaSe_1−*x*_Te_*x*_ nanomaterials undergo hexagonal to monoclinic phase transition as Te content increases, and at magical composition values (0.5 < *x* < 0.7) these two phases start to compete with each other and even coexist to induce unusual band bowing effects and unique multi-phase regions. It is noteworthy to mention that, traditional GaSe–GaTe phase diagram^31^ alone suggests that single-phase GaSe_1−*x*_Te_*x*_ alloying across the full composition is not allowed under equilibrium conditions. Indeed, prior bulk crystal growth of GaSe_1−*x*_Te_*x*_ succeeded in achieving Se-rich and Te-rich composition but composition range 0.25 < *x* < 0.75 remained unknown due to separation into Te-rich and Se-rich regions. Here, we show that full composition in GaSe_1−*x*_Te_*x*_ alloys can be achieved when they are synthesized in nanomaterial form on GaAs (111) substrates through physical vapor transport (PVT) owing to the small lattice mismatch between the substrate and GaSe_1−*x*_Te_*x*_, and possible confinement effects at nanoscales. Observed hexagonal to monoclinic phase crossover and phase coexistence region is strongly evidenced by morphology transformation from isotropic 2D to anisotropic 1D-like features, and proven by Raman spectroscopy, photoluminescence spectroscopy, and high-resolution transmission electron microscopy (HRTEM). Our results provide insight in synthesizing semiconductor alloys far from equilibrium and open-up new opportunities for bandgap engineering through the phase engineering approach, and we predict similar effects in other 2D material systems such as Ti_*x*_Nb_(1−*x*)_X_3_ (X = S,Se) and Mo_*x*_Re_(1−*x*)_S_2_ wherein alloying is attained across two vastly different phases.

## Results

### Synthesis of GaSe_1−*x*_Te_*x*_ in full composition

**Fig. 1:**
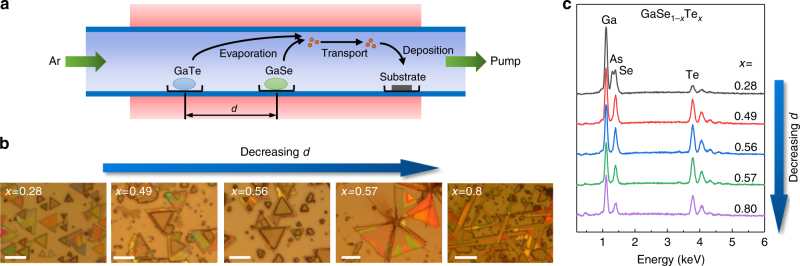
Synthesis process and morphology of GaSe_1−*x*_Te_*x*_ nanostructures. **a** Schematic of the synthesis process of GaSe_1−*x*_Te_*x*_ nanostructures in a single-zone tube furnace. **b** Optical images of the GaSe_1−*x*_Te_*x*_ nanostructures grown at different *d* between GaTe and GaSe source. Corresponding *x* values are shown on the top left corner of each image. Scale bar = 5 μm for *x* = 0.28, 0.49 and 0.56; 10 μm for *x* = 0.57 and 0.8. **c** EDS spectra of the GaSe_1−*x*_Te_*x*_ nanostructures and the calculated composition

The GaSe_1−*x*_Te_*x*_ nanostructures were synthesized by PVT in a single-zone tube furnace using GaSe and GaTe powders as the source materials (Fig. 1a). A detailed description of the growth process can be found in the Methods section. GaAs (111) is chosen as the substrate as it sustains layer-by-layer growth while facilitating epitaxial growth of both GaTe and GaSe owing to the close match in surface symmetry and the inter atomic distance^24, 32, 33^. The composition of the GaSe_1−*x*_Te_*x*_ nanostructures is controlled by the evaporation rate of the GaTe and GaSe source. As shown in Fig. 1a, the GaTe and GaSe sources are separated by a distance *d* with GaSe kept in the center of the furnace and GaTe toward the upstream direction. The evaporation rate of the GaTe source is controlled by positioning it at various temperature regions with different distance *d* from the center. As *d* decreases, the temperature of the GaTe source increases, giving it a higher evaporation rate. At the same time, the GaSe source is fixed in the center so the evaporation rate is kept the same. To study the effect of *d* on the composition of the GaSe_1−*x*_Te_*x*_ nanostructures, we performed EDS measurement on each sample. As shown in Fig. 1c, both Se and Te peaks appear in every sample, indicating the formation of GaSe_1−*x*_Te_*x*_ alloys. The Te content in the GaSe_1−*x*_Te_*x*_ nanostructure increases as *d* decreases, due to the increase of GaTe evaporation rate and partial pressure in the growth chamber.

Interestingly, the morphology of the GaSe_1−*x*_Te_*x*_ nanostructure strongly depends on the Te content in GaSe_1−*x*_Te_*x*_. Closely following from Fig. 1b, on the Se-rich side where *x* < 0.57, the growth typically yields 2D layered triangles with lateral dimension ranging from 1 to 10 μm. This finding agrees well with the prior work on GaSe demonstrating hexagonal phase and threefold symmetry^23, 25^. These triangles are grown in-plane and most of them are aligned along two directions with a 180° angle, indicating the epitaxial growth of the GaSe_1−*x*_Te_*x*_ nanostructures on GaAs (111). At the Te-rich side with *x* > 0.57, however, morphology changes to involve 1D structures (0.57 < *x* < 0.8) which become the dominant morphology when *x* > 0.8. The nanoribbons are also grown in-plane and well aligned along three directions with an angle of 60° between each other when *x* is 0.8. This kind of one dimensional growth is quite similar to our previous study on GaTe^24^, which has a highly anisotropic monoclinic structure that favors to grow along the [010] chain/anisotropy direction. The layered structure and growth direction of the nanoribbon is confirmed by high resolution TEM image and select area electron diffraction (SAED) and taken from a GaSe_1−*x*_Te_*x*_ nanoribbon with *x* = 0.63 as shown in Supplementary Figure 1. These optical images, geometrical anisotropy, and material morphology itself suggests that triangular and 1D-ribbon flakes belong to the threefold symmetry isotropic hexagonal and anisotropic monoclinic phases, respectively, and crossover from 2D to 1D-like features occurs with increasing Te composition. More direct proofs for phase crossover and coexistence of two phases will be discussed within angle resolved Raman and PL spectroscopy as well as HRTEM measurements in the next sections. We note that bulk crystal growth, synthesis, and characterization of GaSe_(1−*x*)_Te_*x*_ alloys have been reported before. In these studies, results have shown that Se-rich (*x* < 0.25) and Te-rich (*x* > 0.75) phases can easily be crystallized but any composition between 0.25 < *x* < 0.75 leads back to Se-rich and Te-rich phases.

### Anomaly in Raman spectrum during phase transition

**Fig. 2:**
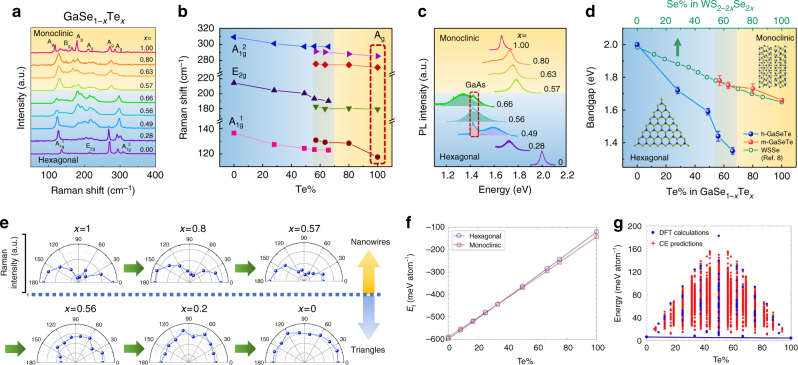
Vibrational properties and bandgap as a function of composition. **a** Raman spectra of GaSe_1−*x*_Te_*x*_ nanostructures at different compositions. Monoclinic and hexagonal structures are separated by different background colors. **b** Peak positions of different Raman modes as a function of Te content in GaSe_1−*x*_Te_*x*_. Blue background indicates the hexagonal side and yellow background indicates the monoclinic side. **c** PL spectra of GaSe_1−*x*_Te_*x*_ nanostructures at different compositions. **d** Bandgap as a function of Te content in GaSe_1−*x*_Te_*x*_. Error bars indicate standard deviation of PL peak position measured from five or more points on the same sample. **e** Evolution of angle resolved Raman peak intensity as Te content decreases. **f**, **g** DFT cluster expansion calculations at a variety of elemental composition shows that there exists a crossover region in formation energies of hexagonal to monoclinic phase GaSeTe systems

To study the hexagonal to monoclinic phase transition in the GaSe_1−*x*_Te_*x*_ nanostructures, we performed detailed Raman and PL studies on these alloys across full composition. Figure 2a shows the Raman spectra of GaSe_1−*x*_Te_*x*_ with x ranging from 0.28 to 0.8, as well as pure GaSe and GaTe for comparison. The figure is separated into two parts—the upper part with yellow background comes from nanoribbons, while the lower part with blue background comes from triangular shaped flakes. At *x* = 0 (pure GaSe), the Raman spectrum contains three peaks at 136 cm^−1^ (A_1g_^1^ mode), 214 cm^−1^ (E_2g_ mode) and 308 cm^−1^ (A_1g_^2^ mode). The two peaks at 268 and 294 cm^−1^ come from the GaAs substrate (Supplementary Figure 2). For *x* ranging from 0.28 to 0.66, the spectra share a similar shape with all three modes of GaSe presenting. A general tendency of softening is observed for all three vibration modes as Te content increases (Fig. 2b), corresponding to larger mass of Te atoms compared to Se. Note that the in-plane E_2g_ mode becomes rather broad and new peaks around it start to emerge as *x* becomes larger than 0.49. This is probably due to an increase in defect density that leads to the breakdown of the selection rule.

As the morphology of the nanomaterial changes from 2D triangle to 1D nanoribbons, a dramatic change in the Raman spectra is observed (Fig. 2a, b). The change is extremely clear when comparing the *x* = 0.56 triangular sample with the *x* = 0.57 nanoribbon. These two samples have very similar Te content but completely different Raman spectra. First, the A_1g_^1^ mode shifts inversely to higher frequencies, and then softens as *x* increases. Second, the GaSe A_1g_^2^ mode disappears and a new peak at 276 cm^−1^ emerges, corresponding to the A_g_ mode at 280 cm^−1^ of pure GaTe. In the monoclinic region, the tendency of softening remains for all vibration modes as Te content increases, consistent with the trend in the hexagonal region. But the sudden change in the spectra characteristics around *x* = 0.6 is a clear sign of hexagonal to monoclinic phase transition. Here, sudden Raman spectrum renormalization is associated with large changes in the crystal structure and symmetry resulting in largely different phonon vibration modes and Raman active modes. Indeed, similar Raman anomalies has been observed in high-pressure driven (diamond anvil cell measurements) phase transition of traditional material systems such as ReS_2_^34^ and Teflon^35^.

### Abnormal band bowing across phase transition region

A more exciting effect of the phase transition comes from the bandgap modulation. Photoluminescence spectra of GaSe_1−*x*_Te_*x*_ measured across the full composition range and the corresponding bandgap values are depicted in Fig. 2c, d, respectively. Here, we note that the GaAs substrate luminescence at 1.42 eV (see Supplementary Figure 2 and highlighted section in Fig. 2c) is fitted by $I = \sqrt {h\nu - E_{\rm{g}}} {\mathrm{exp}}\left[ { - (h\nu - E_{\rm{g}}){\mathrm{/}}k_{\rm {B}}T} \right]$, and the GaSeTe peak is fitted by Gaussian function for accurate determination of the *E*_gap_ values in Fig. 2d. Similar to Raman spectra, an abrupt change in the bandgap is observed as the crystal structure changes from hexagonal to monoclinic. Interestingly, when the composition range is between 0.57 < *x* < 0.67, two different PL peaks, *E*_gap_ values, and phases are simultaneously observed. We refer to this composition range (0.57 < *x* < 0.67) as the structure phase coexistence region. Previously, this kind of phase coexistence behavior has not been observed in any other layered systems or 2D materials such as Mo_*x*_W_(1−*x*)_S_2_, MoS_2*x*_Se_2(1−*x*)_, ReS_2*x*_Se_2(1−*x*)_, and others^8, 11, 13^.

### Isotropic–anisotropic phase crossover and phase coexistence

Potential explanation for this observation could be that two structural phases have rather close formation energies which enables one to attain two phases at a given composition. To offer better understanding to coexisting phases, we have performed density functional theory calculations within cluster expansion formalism (see Methods) to calculate formation energy differences between monoclinic and hexagonal phases at different tellurium content. Results (Fig. 2f-g) shows that initially *x* = 0 (GaSe) and *x* = 1 (GaTe) stabilizes in hexagonal and monoclinic phases respectively, consistent with our results. However, there exists a range of Te% values where two phases become energetically close to each other. For example, when *x* reaches ~40% two phases become energetically degenerate, and thus the two phases can coexist. While theoretically estimated composition range (Te~40%) for coexisting region is close to the experimentally observed values (~55–65%), the differences can be attributed to a variety of factors such as substrate effects, pressure differences (DFT under vacuum vs. experiment under controlled pressure), and inability to account for kinetic/thermodynamic considerations with DFT framework.

We note that the structural phase separation and coexistence observed in GaSeTe is different from the compositional phase separation commonly observed in most alloy systems where full composition alloy is not allowed. In those systems, the material separates into two compositions where alloying is not allowed. This is also true for bulk GaSeTe alloys where compositions of 0.1 < *x* < 0.49 and 0.51 < *x* < 0.85 is inaccessible due to the compositional phase separation as shown in the phase diagram^31^. However, our work shows that when GaSeTe is grown on GaAs (111) in 2D form, those forbidden compositions become accessible for GaSeTe alloys with emergence of structural phase separation and coexistence at the same composition in a certain range.

It is also noteworthy to mention that the band bowing behavior in GaSe_1−*x*_Te_*x*_ (band-gap variation with respect to alloying percentage) is significantly different from other 2D alloy systems owing to the presence of two different competing phases making large changes to the electronic band structure. For example, the WS_2−2*x*_Se_2*x*_ system has similar bandgaps as GaSe_1−*x*_Te_*x*_ at the two ends of *x* = 0 and *x* = 1^8^. The bandgap changes linearly in the WS_2−2*x*_Se_2*x*_ system because the system stays in one phase across full composition range, while it deviates from the linear relationship in the GaSeTe system due to the unique hexagonal to monoclinic phase transition. On the Se-rich hexagonal side as *x* goes from 0→0.66, the bandgap decreases from 2.01 to 1.35 eV, much faster compared to WS_2−2*x*_Se_2*x*_. As *x* changes from *x* = 1→0.67 (the Te-rich side), the band gap increases from 1.66 to 1.78 eV. In these two regions, the bandgap values for single-phase hexagonal (0 < *x* < 0.57) and monoclinic (0.67 < *x* < 1) shows a linear relationship with tellurium content, indicating a small band bowing parameter when the phase remains the same. However, in the phase crossover region, the band bowing theory, which relies on retaining the phase/crystal structure of semiconductor, can no longer be applied as evidenced by the dramatic bandgap change in phase coexistence region. This feature enables wider bandgap tuning range in GaSeTe than WS_2−2*x*_Se_2*x*_. The two-phase coexisting region also allows us to make GaSeTe materials with different bandgaps without changing the composition. Using a linear extrapolation of the band-gap variation across the composition range, we estimate that hexagonal GaTe and monoclinic GaSe (both materials have not been demonstrated before) should possess bandgap at 1.03 and 1.92 eV, respectively.

Concurrent with abnormal band bowing across hexagonal to monoclinic phase transition, synthesized alloys possess unusual isotropic to anisotropic transition. While hexagonal GaSe possesses in-plane isotropy like 2D graphene and MoS_2_, monoclinic GaTe is an anisotropic semiconductor in which atoms are arranged such a way that they form chains running along one particular lattice direction ([010] *b*-axis). To study the effect of phase transition on the structural properties of the GaSe_1−*x*_Te_*x*_ nanostructures, we have employed angle resolved Raman spectra at different Te content as shown in Fig. 2e. Previously, our team and others have successfully utilized angle resolved Raman spectroscopy to determine structural anisotropy direction of variety of 2D materials including monochalcogenides (GaTe)^24, 29^, dichalcogenides (ReS_2_)^36^, and trichalcogenides (MX_3_ M = Hf, Zr, Ti; X = S, Se)^37, 38^. In this method, Raman intensity (*I*_R_) of optical phonon modes involving atomic vibrations (induced polarization *P*_ind_) along the chain (anisotropy) direction is measured as a function of *P* with angle (*α*). When the *P* direction is parallel (perpendicular) to atomic vibrations along (across) chain direction, *P*_ind_ and *I*_R_ is enhanced (reduced). Polar plots (*I*_R_ vs *α*), in return, enable one to determine if material is anisotropic (two-lobed symmetry) and the anisotropy direction (lobe orientation direction).

In our measurements, we have selected optical mode located at 128 cm^−1^ that involves atomic vibrations along the chain direction for angle resolved Raman measurements. Previously, this peak has been successfully used to identify the chain direction of m-phase GaTe, and as expected displays a two-fold symmetry with a period of 180° (Fig. 2e, *x* = 1)^24, 29^. Composition variation across *x* = 1→0 (m-GaTe→ h-GaSe) clearly shows that two-lobed feature turns into more isotropic polar plots in Fig. 2e. Here, we note that almost identical compositions (*x* = 0.56 and 0.57) crystallize in hexagonal and monoclinic phases further proving coexistence of two phases.

**Fig. 3:**
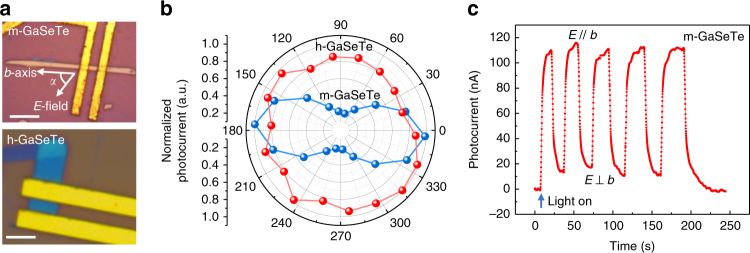
Phase dependent optoelectronic properties. **a** Optical images of the GaSeTe devices based on the monoclinic (top, scale bar = 10 μm) phase and hexagonal (bottom, scale bar = 5 μm) phase. **b** Angle resolved photocurrent as a function of light polarization (*E* field) direction measured on both devices. **c** Time resolved photoresponse of the device based on monoclinic GaSeTe (*V*_ds_ = −0.2 V). The light polarization is alternatively switched between parallel and perpendicular to the *b*-axis

To demonstrate the optoelectronic applications of the unique isotropic to anisotropic phase crossover in GaSeTe alloys, photodetectors based on both monoclinic and hexagonal GaSeTe alloys are fabricated, as shown in Fig. 3a. The phase of the two flakes are identified by Raman measurements based on the above discussion. Angle resolved photocurrent is measured as the polarization (*E* field) direction of the incident light is rotated with respect to the *b*-axis of the crystal. As shown in Fig. 3b, the monoclinic GaSeTe has a clear dichroic response to photons. The photocurrent reaches maximum when the light polarization is parallel to the b-axis and becomes minimum when it is perpendicular to the *b*-axis. This is because the optical absorption coefficient is larger in the parallel setup than the perpendicular setup^24^. The direction dependent photoresponse is a clear demonstration of the anisotropy of the monoclinic GaSeTe. As a distinctive contrast, the hexagonal GaSeTe shows no direction dependent properties, owing to its isotropic hexagonal structure and loss of anisotropy. To further investigate the dichroic response of the monoclinic GaSeTe, we measured time resolved photocurrent change by alternatively switching the light polarization between parallel and perpendicular to the *b*-axis of the crystal. As shown in Fig. 3c, the photocurrent rises rapidly as the polarization becomes parallel to the *b*-axis and drops immediately after turned to the perpendicular direction. The photo-responsivity (*R*) of the m-GaSeTe photodiode was calculated by the formula *R* = *I*_ph_/(*P* × *A*_eff_). Here, *I*_ph_ = |*I*_on_−*I*_off_|, *P* is the incident light power per unit area (488 nm light source at 2.1 mW/cm^2^), *A*_eff_ is the effective illumination area (~5 μm^2^). By calculation, the highest *R* is ~950 A/W at *V*_ds_ = −0.2 V when polarization is parallel to the *b*-axis. In comparison, *R* drops by 5 times down to ~200 A/W at *P*⊥*b*-axis, consistent with our polar photocurrent results.

### Direct observation of hexagonal to monoclinic phase transition

**Fig. 4:**
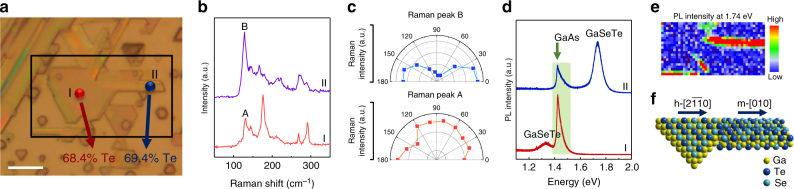
Direct observation of phase transition. **a** Optical image of a GaSe_1−*x*_Te_*x*_ flake with both hexagonal (region-I) and monoclinic (region-II) phase grown simultaneously. The composition for each region is indicated by Te% in the figure. Scale bar = 5 μm. **b** Raman spectra taken at region-I and region-II from **a. c** Angle resolved Raman peak intensity of peak A and B from region-I and II. **d** PL spectra taken at region-I and II. GaAs substrate peak is labeled by green background. **e** PL mapping of the emission intensity at 1.74 eV taken from the area indicated by the rectangular in **a**. **f** Schematic of the 3D atomic structure of the coexisting hexagonal and monoclinic phases

In Fig. 4, we provide further proof that these vastly different phases coexist simultaneously within the same flake in phase separated form. This is in contrast to any other data sets in Fig. 2 where different phases were observed at same compositions but on different flakes. A closer look at Fig. 4a demonstrates the presence of triangular and nanoribbon features: despite the dramatic difference in the morphology, both regions have close Te content—68.4% for triangles and 69.4% for the nanoribbons. The SEM image and EDS spectra of the two regions can be found in Supplementary Figure 4. Raman spectrum and angle resolved Raman data sets in Fig. 4b, c clearly demonstrate phase separated hexagonal (region-I red) and monoclinic (region-II blue) phases. These two phases are clearly distinguished by PL spectra as shown in Fig. 4d. The hexagonal region-I has a PL peak at 1.34 eV, while the monoclinic region-II shows a peak at 1.74 eV. Both PL emission energies fit into the linear bandgap relation vs Te content as discussed in Fig. 2d. Note that 1% minuscule composition difference across these two regions leads to colossal bandgap renormalization of 0.4 eV. Coexisting phases on the same flake is best observed from PL mapping of the 1.74 eV peak intensity in Fig. 4e. PL mapping of 1.74 eV peak shows that PL intensity is maximized around needle like nanoribbons. Based on this we have constructed 3D view of the coexisting phase interfaces/boundaries in Fig. 4f. It is noteworthy to point out that the PL intensity increases suddenly as it goes from region-I to region-II, indicating the interface between the two phases is sharp without any gradual transition area. This sample with mixed phases is grown at a lower pressure (250 Torr) than the previous sample (300 Torr) with dominant monoclinic phases. The effect of growth pressure and growth mechanism of this mixed phase flake is discussed in the Supplementary Discussion and Supplementary Figure 3 and Supplementary Figure 4.

### Atomic scale insights into phase separation

**Fig. 5:**
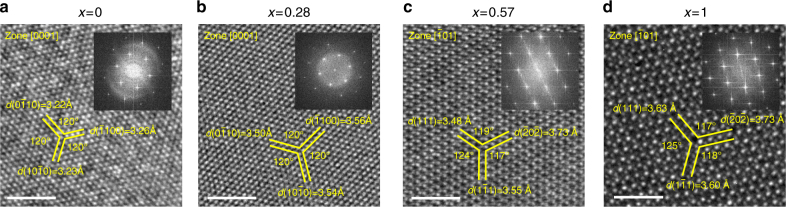
Evidence of phase transition from TEM. TEM and FFT patterns for GaSe_1−*x*_Te_*x*_ with **a** *x* = 0, **b** 0.28, **c** 0.57, and **d** *x* = 1. The zone axis, interplanar distance, and angle between planes are indicated in each image. Scale bars = 2 nm

The crystal structure of both the hexagonal and monoclinic phases are studied by HRTEM. Figure 5 shows the TEM images and corresponding fast Fourier transform (FFT) patterns for GaSe_1−*x*_Te_*x*_ with *x* = 0, 0.28, 0.57, and 1. For the Se-rich *x* = 0 and 0.28 samples, a six-fold symmetry is revealed with the angles between the ${\mathrm{(10}}\bar 1{\mathrm{0),}}\,{\mathrm{(}}\bar 1{\mathrm{100)}}\,{\mathrm{and}}\,{\mathrm{(0}}\bar 1{\mathrm{10)}}$ planes all being 120° as shown in Fig. 5a, b. The interplanar distance for the three planes closely match each other with very small difference between the maximum (*d*_max_) and minimum (*d*_min_) measured values (0.04 Å for *x* = 0 and 0.06 Å for *x* = 0.28). These features agree well with the characteristics of the hexagonal crystal system. Note that the interplanar distance increases as *x* increases from 0 to 0.28, because of the incorporation of the larger Te atoms compared to Se. For the Te-rich *x* = 0.57 and *x* = 1 samples, a completely different structure is found as shown in Fig. 5c, d. First, the six-fold symmetry is lost and a two-fold symmetry emerges, with the angles between the $\left( {{\mathrm{111}}} \right){\mathrm{,}}\,\left( {\bar 20\bar 2} \right){\mathrm{,}}\,{\mathrm{and}}\,\left( {1\bar 11} \right)$ planes deviate from 120°. Second, the three planes possess different interplanar distance, with the *d*_max_−*d*_min_ being 0.25 Å for *x* = 0.57 and 0.33 Å for *x* = 1. The interplanar distance of the GaTe sample (*x* = 1) agrees well with the data of monoclinic GaTe from the ICDD database (PDF card No. 44-1127). These findings confirm the monoclinic structure of the *x* = 0.57 and *x* = 1 samples.

## Discussion

Our results demonstrate phase crossover and anomalous band bowing effects in GaSe_1−*x*_Te_*x*_ nanostructures when the composition is continuously varied across two completely different materials, namely isotropic hexagonal GaSe (*x* = 0) and anisotropic monoclinic GaTe (*x* = 1). While traditional GaSe–GaTe phase diagram^31^ alone suggests that single-phase GaSe_1−*x*_Te_*x*_ alloying across the full composition is not possible, results herein show that GaAs (111) substrate plays an essential role in stabilizing the metastable phases and enable wide composition variation to access previously unavailable phase crossover region. We also discussed the effect of different substrates including highly oriented pyrolytic graphite and c-cut sapphire and the details can be found in the Supplementary Discussion and Supplementary Figure 5. As tellurium is alloyed into GaSe (increasing *x* concentration), GaSe_1−*x*_Te_*x*_ nanostructures undergo hexagonal to monoclinic and isotropic to anisotropic phase crossover at *x* = 0.5–0.7. Interestingly, formation energies of these two phases become comparable in composition range at 0.56 < *x* < 0.66, consequently they start compete with other yet both of these two phases coexist simultaneously. Angle resolved Raman, Raman spectroscopy, optical images, PL spectroscopy, and HRTEM measurements all prove the phase crossover as well as the presence of coexisting phases. Because of large competition between hexagonal and monoclinic phases, GaSe_1−*x*_Te_*x*_ nanostructures display discontinuity and large band bowing effects across the composition range that has not been witnessed in any other layered or 2D systems. This abnormal band bowing effect allows us to obtain a bandgap tuning range of 1.35 to 2.01 eV, which is larger than the mostly studied TMDC alloy systems in the 2D materials family^6, 8, 12, 39^. This approach has the potential to be generalized to other alloy systems with the two components belonging to different phases. By controlling the kinetic factors such as growth pressure, a two-phase region with both hexagonal and monoclinic phase coexisting can be created. This enables us to form GaSe_1−*x*_Te_*x*_ alloys with the same composition but completely different crystal structures and properties, such as bandgap and anisotropy. Since both phases can be synthesized on a single wafer simultaneously in a single growth process, we can use the flake of either phase on demand. This facilitates multi-wavelength light emission and absorption applications from this material at the same composition. The coexistence of the isotropic hexagonal phase and the anisotropic monoclinic phase also opens opportunities to make GaSeTe nanomaterials with different response to polarized light without changing the composition. For example, at 60% Te composition, both isotropic and anisotropic phases coexist. Each of these phases not only have different band gaps but also different optical responses to linearly polarized light. This means one phase can be selectively activated on demand (by wavelength or polarization direction), leading to selective material response behavior. The phase transition on a single flake also has great potential in fabricating high quality GaSe_1−*x*_Te_*x*_ heterojunctions with sharp interfaces. Overall, our results deepen our understanding for the gallium chalcogenide family and provide guidelines for tuning material properties through phase engineering.

## Methods

### Materials synthesis

The PVT synthesis of GaSe_1−*x*_Te_*x*_ nanostructures was carried out in a tube furnace with a 1″ quartz tube. GaSe (60 mg) and GaTe (20 mg) powders (American Elements) were used as the source materials and Ar was used as the carrier gas. Bare GaAs (111) wafers were used as is for growth substrates. The GaSe and GaTe powders were loaded in two quartz boats and sent into the tube. The GaSe boat was put in the center of the tube and the GaTe boat was located upstream from GaSe. The distance between GaTe and GaSe was set in the range of 7–16 cm. The substrate was located 15 cm away downstream. The tube was evacuated to 10 mTorr and then heated from room temperature to 780 °C with a ramping rate of 20 °C/min. The temperature was kept at 780 °C for 5 min and then cooled down to room temperature. The Ar flow rate was set at 50 sccm and the growth pressure was 300 Torr for the whole process. For the mixed phase flake the pressure was set at 250 Torr. The pure GaSe and GaTe bulk crystals were synthesized by modified Bridgman growth technique^40^ in a single-zone furnace at temperatures ranging from 850 to 1020 °C for three weeks.

### Materials characterization

Room temperature PL and Raman measurements for the GaSe_1−*x*_Te_*x*_ nanostructures were performed in a Renishaw InVia spectroscopy system with a ×100 objective lens using a laser source of 488 nm wavelength. The laser was focused onto the sample with a spot diameter of 0.5 µm. Angle resolved measurements were carried out in the same system by mounting samples on a rotation stage and taking data when the sample is rotated every 20°. The incident laser and detector were polarized parallel to each other along the 0–180° direction. SEM and EDS measurements were performed on a Hitachi S4700 field emission SEM with a working distance of 12–15 mm and acceleration voltage 15 kV. TEM samples were prepared by dispersing the GaSe_1−*x*_Te_*x*_ flakes in isopropyl alcohol (IPA) through sonication and dropping the IPA onto copper grids with holey carbon films. TEM and SAED measurements were performed on the FEI Titan TEM operated at 300 kV.

For the device fabrication, the flake was de-cleaved from GaSeTe samples grown onto GaAs substrates. Substrates were first indented by diamond tips of ~5 µm in diameter to delaminate samples lightly. Samples were mildly sonicated to release GaSeTe sheets that are transferred onto 285 nm SiO_2_/Si substrates using a mechanical transfer station. The 5 nm Ti/50 nm Au electrode is deposited onto the substrate by standard electron beam lithography. A 488 nm light source at 2.1 mW/cm^2^ power density is used for the photodetector characterization.

### DFT calculations

We used the Vienna Ab initio Simulation Package (VASP)^41^ for density functional theory calculations. To simulate hexagonal and monoclinic GaSe_1−*x*_Te_*x*_ different compositions (*x* = 0, 1/12, 2/12, 3/12, 4/12, 6/12, 8/12, 9/12, and 1), we used 3×3×1 and 1×3×1 supercells, respectively, leading to 72 atoms in each supercell. The supercell structures were generated using the method based on the Special Quasirandom Structures (SQS) method^42^ implemented in the ATAT package^43^. In the VASP calculations, we used the Perdew-Burke-Ernzerhof functional^44^ and potentials from the projector augmented-wave method^45, 46^. Plane-wave basis set with a cutoff energy of 500 eV were used and the *k*-point sampling grids for the supercells were set to 4×4×2 and 2×6×2 for the hexagonal and monoclinic supercells, respectively. The formation energy *E*_f_ of GaSe_1−*x*_Te_*x*_ is defined as *E*_f_ = (*E*_GaSe1−*x*Te*x*_−*E*_Ga_−(1−*x*)*E*_Se_−*xE*_Te_)/2, where *E*_GaSe1-*x*Te*x*_ refers to the energy of a GaSe_1−*x*_Te_*x*_ supercell per formula unit. *E*_Ga_, *E*_Se_, and *E*_Te_ denote the energies of Ga, Se, and Te atoms in their corresponding bulk unit cells. We also used the cluster expansion method in ATAT to search for possible ordered compounds with the compositions lying between hexagonal GaSe and GaTe.

### Data availability

The data that support the findings of this study are available from the authors on reasonable request.

## Electronic supplementary material

Supplementary Information
