# Supplementary material for: Abnormal band bowing effects in phase instability crossover region of GaSe1-xTex nanomaterials
Source: Nat Commun. 2018 May 15;9:1927. doi: 10.1038/s41467-018-04328-z (PMC5953935; doi:10.1038/s41467-018-04328-z)
Supplement: Supplementary file 1 — Supplementary Information [file 41467_2018_4328_MOESM1_ESM.pdf]

Supplementary Information

Title: Abnormal Band Bowing Effects in Phase Instability Crossover Region of $GaSe_{1-x}Te_x$ Nanomaterials

Cai et al.

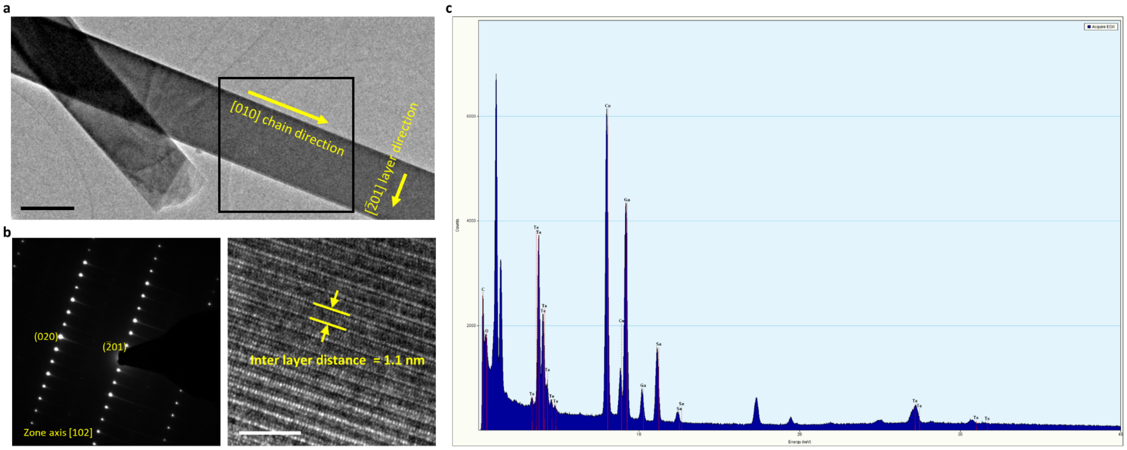

Supplementary Figure 1 | a. Low magnification TEM image of a $GaSe_{1-x}Te_x$ nanobelt with x=0.63. Scale bar=0.5 μm. **b.** SAED (left) and high resolution TEM image (right) taken from zone axis [102] in the area indicated by the rectangular in (**a**). Scale bar=5 nm. **c.** EDS spectrum of the $GaSe_{1-x}Te_x$ nanobelt.

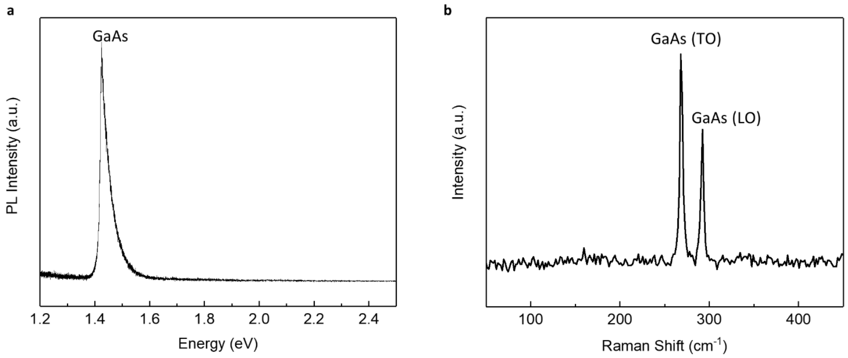

Supplementary Figure 2 | a. PL spectrum of the GaAs (111) substrate. **b.** Raman spectrum of the GaAs (111) substrate showing the transverse optical (TO) mode and the longitudinal optical (LO) mode.

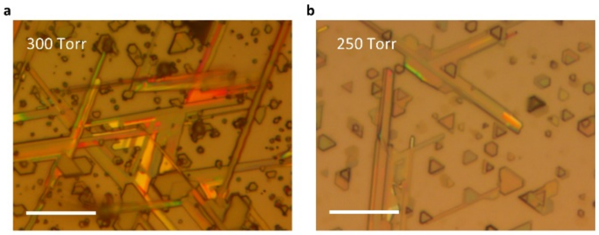

Supplementary Figure 3 | Optical images of GaSeTe nanomaterials grown at 300 Torr (**a**) and 250 Torr (**b**). Scale bars=10 μm.

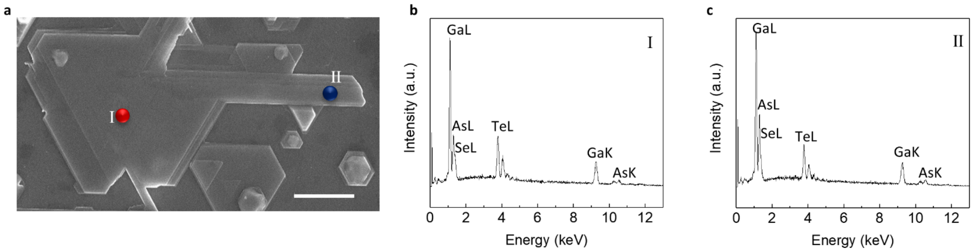

Supplementary Figure 4 | a. SEM image of the flake with coexisting hexagonal and monoclinic phase. Scale bar=2 μm. **b-c.** EDS spectra taken from region I and II indicated in **a**.

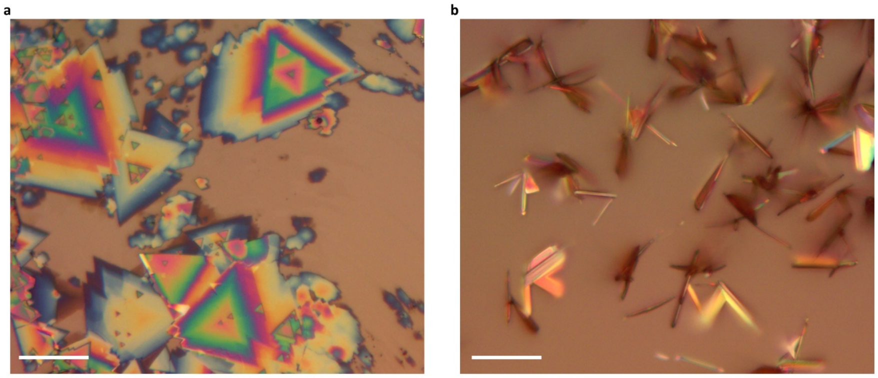

Supplementary Figure 5 | Optical images of GaSeTe nanomaterials grown on HOPG (**a**) and sapphire (**b**). Scale bars=10 μm.

Supplementary Discussion

Effect of Growth pressure. Supplementary Figure 3 shows the effect of growth pressure on the GaSeTe nanomaterials grown on GaAs (111). Both samples are grown at *d*=7 cm, same as the x=0.8 sample. At 300 Torr, the dominate structure of GaSeTe is nanobelts with monoclinic phase, while as the pressure decreases to 250 Torr, more triangles with hexagonal phase start to emerge. A possible explanation is that as the total pressure is decreased, the reaction becomes more transport-limited, leading to a lower deposition rate which facilitates layer by layer (LBL) growth. Furthermore, lower deposition rate gives the atoms more time to move freely on the GaAs(111) surface which has similar symmetry as the hexagonal phase. As a result, the hexagonal phase can be stabilized at lower growth pressure.

Growth mechanism of the mixed phase flake. As discussed in the manuscript, region-I in Supplementary Figure 4 is assigned to the hexagonal phase and region-II belongs to the monoclinic phase. In this flake, the triangle-like region in labeled as I and the sticking out nanobelt is labeled as II. This sample is the same one as shown in Supplementary Figure 3b. We believe that in this mixed phase flake, the triangle region nucleates and grows on the GaAs (111) surface epitaxially first, followed by the growth of the nanobelts extending outward. The size of the hexagonal phase is limited due to the strain induced by the lattice mismatch between $GaSe_{1-x}Te_x$

and the GaAs substrate. As the hexagonal phase grows larger, it becomes less stable and the LBL growth turns into dendritic growth, transforming the hexagonal phase to monoclinic phase characterized by the nanobelt shape in region-II.

Effect of different substrates. To elaborate the effect of substrates on the phase instability of the GaSeTe nanomaterials, we performed similar growth process on different substrates other than GaAs (111), including c-cut sapphire and highly oriented pyrolytic graphite (HOPG). The growth condition is the same as It can be seen that the phase of the GaSeTe nanomaterials strongly depends on the choice of the substrate. On HOPG, all flakes are grown in triangle shapes with hexagonal phase; while on sapphire, all flakes are grown in nanobelts with monoclinic phase.

This dramatic difference can be explained by the different surface structure of the substrate and different interaction mechanism between the substrate and the GaSeTe nanomaterials. The surface of HOPG has a atomic flat hexagonal structure formed by carbon atoms arranging in honeycomb shape. As compared to the GaAs (111) surface, this structure is more isotropic and more similar to the surface of the hexagonal GaSeTe. This enables isotropic propagation of the GaSeTe adatoms on the HOPG surface and stabilization of the hexagonal structure of the GaSeTe nanomaterials[1]. On sapphire, most of the GaSeTe flakes are grown out of plane and belong to the monoclinic phase. This can be explained by the large lattice mismatch between sapphire and GaSeTe, which suppresses in-plane epitaxial growth. As there is no constraining force from the substrate, the Te-rich GaSeTe grows in its thermodynamically stable phase which is the monoclinic phase.

Supplementary References:

1. Li X*, et al.* Van der Waals Epitaxial Growth of Two-Dimensional Single-Crystalline GaSe Domains on Graphene. *ACS Nano* **9**, 8078-8088 (2015).
